# Improving comfort in people with dementia and pneumonia: a cluster randomized trial

**DOI:** 10.1186/s12916-016-0663-x

**Published:** 2016-08-11

**Authors:** Tessa van der Maaden, Henrica C. W. de Vet, Wilco P. Achterberg, Froukje Boersma, Jos M. G. A. Schols, David R. Mehr, Francisca Galindo-Garre, Cees M. P. M. Hertogh, Raymond T. C. M. Koopmans, Jenny T. van der Steen

**Affiliations:** 1 EMGO Institute for Health and Care Research, VU University Medical Center, Amsterdam, The Netherlands; 2 Department of General Practice & Elderly Care Medicine, VU University Medical Center, Amsterdam, The Netherlands; 3 Department of Epidemiology and Biostatistics, VU University Medical Center, Amsterdam, The Netherlands; 4 Department of Public Health and Primary Care, Leiden University Medical Center, Leiden, The Netherlands; 5 Department of General Practice, Elderly Care Medicine, University Medical Center Groningen, Groningen, The Netherlands; 6 Department of Family Medicine and Department of Health Services Research, Maastricht University, Maastricht, The Netherlands; 7 Department of Family and Community Medicine, School of Medicine, University of Missouri, Columbia, MO USA; 8 Department of Primary and Community Care, Radboud University Medical Centre, Nijmegen, The Netherlands; 9 Joachim en Anna, center for specialized geriatric care, Nijmegen, The Netherlands; 10 Radboudumc Alzheimer Center, Nijmegen, The Netherlands

**Keywords:** Nursing homes, Dementia, Pneumonia, Discomfort, Practice guideline, Trial

## Abstract

**Background:**

Pneumonia in people with dementia has been associated with severe discomfort. We sought to assess the effectiveness of a practice guideline for optimal symptom relief for nursing home residents with dementia and pneumonia.

**Methods:**

A single-blind, multicenter, cluster randomized controlled trial was conducted in 32 Dutch nursing homes. Outcomes were assessed on the patient level. The main outcome measures were discomfort and symptoms: discomfort (DS-DAT: Discomfort Scale-Dementia of Alzheimer Type), (lack of) comfort (EOLD-CAD: End Of Life in Dementia-Comfort Assessment in Dying), pain (PAINAD: Pain Assessment in Advanced Dementia), and respiratory distress (RDOS: Respiratory Distress Observation Scale). Outcomes were scheduled daily from diagnosis until 10 days later and a final time between 13–15 days from diagnosis by trained observers who were blinded to the intervention and the residents’ condition and treatment. In a pre-intervention phase, usual care was provided to all homes. In the intervention phase, matched clusters of homes were randomized to either the control (*n* = 16) or intervention condition (*n* = 16).

**Results:**

Between 1 January 2012 and 1 May 2015, 464 episodes of pneumonia were included. Outcomes were obtained for 399 episodes in 367 residents. Longitudinal multilevel linear regression analyses were performed on log-transformed outcomes, so coefficients should be interpreted as a ratio, and a coefficient of 1 means no difference. The practice guideline in the intervention phase did not reduce the level of discomfort and symptoms: DS-DAT: 1.11 (95 % CI 0.93–1.31), EOLD-CAD: 1.01 (95 % CI 0.98–1.05), PAINAD: 1.04 (95 % CI 0.93–1.15), RDOS: 1.11 (95 % CI 0.90–1.24). However, in both the intervention and control groups, lack of comfort and respiratory distress gradually decreased during the entire 3.5 years of data collection, and were lower in the intervention phase compared to the pre-intervention phase: DS-DAT: 0.93 (95 % CI 0.85–1.01), EOLD-CAD: 0.98 (95 % CI 0.97–1.00), PAINAD: 0.96 (95 % CI 0.91–1.01), RDOS: 0.92 (95 % CI 0.87–0.98).

**Conclusions:**

When compared to usual care, the practice guideline for optimal symptom relief did not relieve discomfort and symptoms in nursing home residents with dementia and pneumonia. However, discomfort and symptoms decreased gradually throughout the data collection in both the intervention homes and the control homes. An intervention that focuses on creating awareness may be more effective than a physician practice guideline.

**Trial registration:**

The Netherlands National Trial Register (ID number NTR5071. Registered 10 March 2015).

**Electronic supplementary material:**

The online version of this article (doi:10.1186/s12916-016-0663-x) contains supplementary material, which is available to authorized users.

## Background

Nursing home residents with dementia and pneumonia experience severe discomfort which increases in the six days preceding death [1, 2]. Discomfort occurs regardless of treatment with antibiotics or not. Residents dying from pneumonia experienced more discomfort than residents dying of other causes [3]. Moreover, death from respiratory infections has been associated with the largest symptom burden before death. For example, 78 % experienced respiratory distress compared to 40 % and 23 % for residents dying from a cardiovascular disorder or dehydration/cachexia, respectively [4]. Nowadays, comfort is increasingly accepted to be a primary goal of treatment for residents with dementia, especially — but not exclusively — for those nearing death. However, evidence on the best way to maximize comfort for these residents is scarce.

Because residents with dementia are often unable to express their complaints and wishes about treatment, relieving the symptoms of pneumonia in these residents is particularly challenging, and palliative care guidelines directed towards the treatment of symptoms in other populations lack applicability. In contrast to chronic progressive diseases such as cancer, for which guidelines are developed, pneumonia is an acute intercurrent disease with specific symptoms. Until recently, no specific guidelines were available, and no studies have tested evidence-based recommendations to intervene in usual care to relieve discomfort in residents with dementia and pneumonia.

We developed a practice guideline for optimal relief of the symptoms of pneumonia specifically for residents with dementia, based on existing guidelines, the available literature, and consensus among a multidisciplinary and international expert panel in a Delphi study [5]. This practice guideline provides targeted treatment recommendations and a checklist including observational instruments for the monitoring of pneumonia symptoms. We hypothesized that the practice guideline would enhance comfort by regular observations to monitor symptoms, by providing a more structured treatment approach and also by increasing awareness of discomfort. We assessed the effects of the introduction of this evidence- and consensus-based practice guideline on the level of observed discomfort, (lack of) comfort, pain, and respiratory distress (in brief: discomfort and symptoms) from the diagnosis of pneumonia until cure or death within 15 days. We compared the practice guideline with usual care (randomized part of the study) but also over time (comparison over time).

## Methods

### Design, setting, and inclusion

We conducted a single-blind, multicenter cluster randomized controlled trial in 32 nursing homes (the clusters) covering 11 of 12 provinces from January 2012 until May 2015. The cluster randomized design was chosen so that physicians would not have patients randomized to use of the guideline and non-use of the guideline, as physicians could always use the guideline once familiar with it. The trial period comprised a pre-intervention phase (before randomization) and an intervention phase (after randomization) to allow for adjusting for changes in the outcomes over time. Dutch nursing homes employ elderly care physicians, who are responsible for all medical care and treatment decisions after having followed a 3-year specialist training in elderly care medicine that includes, for example, training in advance care planning and decision making in end-of-life care [6–8]. Based on a clinical diagnosis (pneumonia as most probable diagnosis), attending physicians included all episodes of pneumonia (multiple episodes per resident possible) in residents with dementia who resided on wards that participated in the study.

### Outcome measures

The study’s primary outcome measures were discomfort and important symptoms for persons with pneumonia: pain and respiratory distress. Improving comfort was the primary goal of the intervention, and it may be the primary care goal for patients with advanced dementia. Pain and respiratory distress were also assessed, as these are burdensome symptoms and are commonly experienced by patients with dementia in the dying phase [4, 8, 9]. We assessed residents’ discomfort using the validated Discomfort Scale-Dementia of Alzheimer Type (DS-DAT) [10, 11]. Additionally, we measured (lack of) comfort with the End Of Life in Dementia-Comfort Assessment in Dying (EOLD-CAD) [12, 13]; pain, using the Pain Assessment in Advanced Dementia (PAINAD) [14, 15]; and respiratory distress with the Respiratory Distress Observation Scale (RDOS) [16, 17]. These observational instruments are described in more detail elsewhere [1].

The level of sleepiness was also observed, because being unconscious or asleep may positively affect comfort [1]. A six-level scale (“awake and alert,” “awake,” “awake but sleepy,” “falling asleep,” “in a light sleep,” and “in a deep sleep”) was used as a continuous scale in analyses. Additionally, we registered the use of visible non-pharmacological measures such as extra pillows to improve posture or oxygen administration.

### Data collection

Observers who did not know the residents or the unit they visited performed the observations, to ensure they remained blind to the residents’ condition, treatments, and the intervention. Observers had various backgrounds [1]; some were nursing home staff working on wards not participating in the study, and others, such as BSc students, had no relationship with the nursing home. The research team trained all 229 observers (mean 7, range 1–14 per home) in using the observational instruments using the same program that included an instructional video and training with videotaped residents to increase the reliability of observations.

**Fig. 1:**
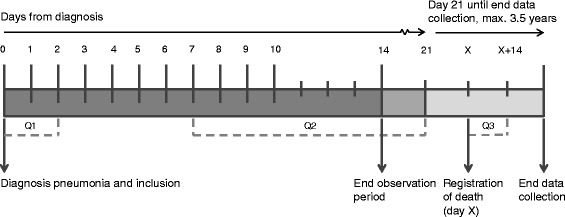
Timeline of data collection. *Q1* questionnaire 1, *Q2* questionnaire 2, *Q3* questionnaire 3, *X* day of death. Observers were scheduled to assess outcomes on day 0 and day 1 (2 times a day), daily for days 2–10 and once on day 13, 14, or 15. Attending physicians included residents at the time of diagnosis of pneumonia, completed Q1 within 2 days from diagnosis, Q2 between 7 days and 21 days from diagnosis, and Q3 when a resident died during the data collection within 14 days from (registration of) death. Data analyzed for the purpose of this article only consider observational data, inclusion of Q1 and Q2, and survival

Observations of all outcomes preferably started at the day of diagnosis (further referred to as day 0), and from then on observations were planned twice a day at day 0 and day 1, once a day from day 2 until day 10, and one last time at day 13, 14, or 15 (Fig. 1). We limited the total observation period to 15 days because cure from pneumonia or death were both expected by this time [2]. Observations were scheduled at roughly the same times each day, and not during meals or shortly after potentially burdensome procedures such as transfers, washing, or toileting. Each observation took approximately 10 minutes.

We allowed fewer than the maximum number of 13 observations, prioritizing regular observations, i.e., avoiding consecutive missed observations. Reasons for missing observations were a limited availability of trained observers, e.g., during the weekends, and late inclusion by the attending physician (i.e., after the day of diagnosis). We had regular scheduled e-mail contact with the observers in all homes during the study period to discuss any problems in data collection and ways to resolve them.

### Other measures and treatments

At the time a pneumonia episode was included (Fig. 1), the attending physician provided demographics, specified whether the resident had a treatment goal primarily directed at comfort (comfort goal) or otherwise (cure or life prolongation), and provided a clinical judgment of illness severity (scale ranging from 1 (not ill) to 9 (moribund)) [18]. Questionnaire 1 (Q1), completed within 2 days from the diagnosis of pneumonia, addressed symptoms of pneumonia at time of the diagnosis and treatments initiated to relieve symptoms. In questionnaire 2 (Q2), completed between 1 and 3 weeks from diagnosis, physicians provided the following: treatments initiated to relieve symptoms at follow-up, the severity of dementia using the 7-item Bedford Alzheimer Nursing Severity Scale (BANS-S) [19], whether there was full dependency on seven activities of daily living (ADL) items (dressing, transfer, eating, toilet use, personal hygiene, bed mobility, locomotion on unit) [20], and degree of dependency in three distinct activities (dressing, walking, and eating), each assessed on a 5-point scale. Physicians also provided data about urinary incontinence, comorbid diseases, nutritional and hydration status, and delirium as judged by the physician. To estimate the risk of dying within 2 weeks when treated with antibiotics, we used a validated 8-item prognostic score (range 0–31) [21].

### Randomization

Nursing homes (clusters) started with the pre-intervention phase of the study, in which care as usual was provided. During this pre-intervention phase, homes were asked to include residents with pneumonia, who were observed following the observation protocol, while physicians provided data about the residents’ condition and treatments as described previously. After a minimum of 6 months, a maximum of 24 months, and after reporting at least four residents with pneumonia, participating nursing homes were randomized. One year before the end of the data collection (May 2014), the last 11 homes were randomized even if homes had included only one or two residents in the pre-intervention phase.

Within groups of homes, two smaller groups that were comparable with regard to (1) the number of psychogeriatric residents (predominantly residents with dementia), (2) the location in the urbanized central west region of the country or elsewhere, and (3) the level of discomfort measured in the pre-intervention phase (average DS-DAT score on observation day 2) were randomized to either the control group or intervention group by drawing lots in opaque, sealed envelopes. After the randomization procedure was finished, the total number of beds was similar in the control group (16 homes) and the intervention group (16 homes) (control 1652 (mean 103), intervention 1700 (mean 106)). Likewise, the number of homes located in the urbanized west region of the country (control 10, intervention 9) and pre-intervention discomfort (mean DS-DAT score: control 8.2, intervention 8.2) were not different.

### The intervention

The contents and key points of the practice guideline are listed in Table 1; more details on its contents and development are described elsewhere [5]. Practice guideline components were (1) a checklist with symptoms of pneumonia, (2) observational instruments to monitor the symptoms of pain and respiratory distress, and (3) tailored treatment recommendations about supportive care, non-pharmacological treatment, and pharmacological treatment for nine symptoms related to pneumonia (Table 1). Physicians were instructed to use the different components of the practice guideline at their own discretion. Using the guideline was not mandatory, so we were unable to assess physician’s adherence to specific guideline recommendations. Our recommendations for use were to: (1) thoroughly read the practice guideline at least once before treating or including patients, (2) for a specific patient, use the checklist to monitor symptoms and assess symptoms at diagnosis, use it daily on the two days after diagnosis, and one last time seven days after diagnosis, (3) use observational instruments in the case of suspected respiratory distress or pain, and (4) use the tailored treatment recommendations in the guideline in response to the completed checklist or the patient’s condition.

**Table 1:** Contents of the practice guideline

The intervention		
A consensus- and literature-based practice guideline for optimal symptom relief for residents with pneumonia and dementia		
Practice guideline components:		
1. Checklist of symptoms		
2. Observational instruments to monitor symptoms*		
3. Tailored treatment recommendations		
4. Poster displaying key points and action plan		
*Respiratory distress	RDOS:	Respiratory Distress Observation Scale
*Pain	PAINAD:	Pain Assessment in Advanced Dementia
	PACSLAC:	Pain Assessment Checklist for Seniors with Limited Ability to Communicate
	REPOS:	Rotterdam Elderly Pain Observation Scale
Action plan:		
A. Suspected pneumonia in resident with pneumonia and dementia		
B. Right away: complete checklist		
C. Optional: use the RDOS to asses respiratory distress		
D. Optional: observe pain using one of three instruments		
E. Consult relevant treatment recommendations		
F. Re-use the checklist on days 1, 2, and 3 and 7 days after pneumonia diagnosis		
G. Repeat steps at a later time and monitor symptoms using the checklist and observational instruments		
Key points:		
- Treatment advice is grouped into supportive care and (non)pharmacological treatments		
- The practice guideline is suitable for all treatment goals, including cure or palliation		
- The practice guideline is intended as a decision aid; the physician is of course free to deviate from it if there are good clinical reasons		
- Administering a low dose of opioids can provide relief of the overall condition of the resident, regardless of the treatment goal		
The practice guideline was expected to enhance comfort by:		
- Enhancing awareness with regard to discomfort		
- Providing a more structured treatment approach		
- Ensuring regular observations to monitor symptoms		

The intervention was introduced in all intervention homes during a 1-hour meeting at the start of the intervention phase, and physicians were supposed to apply it with individual patients. During the study period, physicians were reminded to use the practice guideline by monthly reminder e-mails, a semi-annual newsletter, and regular phone calls. Physicians were instructed to use the practice guideline and its components at their own discretion. In the control homes, residents with dementia and pneumonia received usual care to relieve symptoms. At the transition to the intervention phase in control homes, an evaluation meeting was organized, in which we addressed the assignment to the control group, the study’s progress in general, and the process in the particular nursing home. Control homes were told that an intervention for optimal symptom relief was introduced in the intervention homes, but they were not informed about its contents, format, or possible mechanisms to enhance comfort.

### Sample size

We based the sample size calculations on the number of pneumonia episodes rather than on the repeated observations within a pneumonia episode. The time point on which we could observe the largest difference in discomfort between the control group and the intervention group was not determined in advance, because the exact day on which the maximum effect is expected depends on several factors, such as severity of illness, the moment (symptom-relieving) treatment was started (directly after pneumonia diagnosis or later), and what treatment was initiated. When symptom-relieving treatments such as analgesics are provided, effects on observed discomfort are expected soon after administration. With antibiotics, relief of symptoms, if any, would be expected from about 48 hours after administration. Further, we chose the outcome measure discomfort (DS-DAT) as a basis, and used an estimated incidence of pneumonia of 0.095 episode per psychogeriatric bed per year [22]. We assumed nursing homes would participate with an average of 125 beds on psychogeriatric wards (mostly dementia) and that observations were started on time for five of six episodes of pneumonia. Based on these assumptions, a maximum intracluster correlation of 0.20, a significance level (α) of 0.05 (two-tailed), and a power (1 – -β) of 0.80, at least 12 homes per group were needed for the DS-DAT, 5 homes for the PAINAD, and 20 homes for the EOLD-CAD to detect a clinically relevant difference at some days of the observation period between the intervention homes and the control homes of 3 points on the DS-DAT (assuming a standard deviation (SD) of 2.98) [23] and the EOLD-CAD (assuming an SD of 3.43) [12] and 1.5 points on the PAINAD (assuming an SD of 1) [15]. The mean values were robust estimates, as they were based on 3, 4, and 2 observations, respectively.

### Analyses

We calculated the incidence of pneumonia by summing incident cases of pneumonia during the months that homes participated in the study. We included data on “missed episodes”: pneumonia episodes that met the inclusion criteria but were not included in the study, as reported retrospectively by the physicians. Descriptive statistics were used for resident characteristics, symptoms of pneumonia, and treatments to relieve symptoms. To compare residents between and within groups, independent *t* tests, chi-square tests, and a Mann–Whitney *U* test (length of stay) were used.

We used longitudinal multilevel linear regression analyses and added the binary variable intervention phase (0 = pre-intervention phase, 1 = intervention phase) to the model, as well as an interaction term for intervention phase and group (control or intervention). We modeled the outcome variables as a function of group (control or intervention) and intervention phase (pre-intervention phase or intervention phase) to account for pre-intervention differences between the groups. The intervention effect is reflected by the difference between intervention homes and control homes in the intervention phase, adjusted for the pre-intervention phase. We used an additional longitudinal multilevel linear regression model to assess differences between the pre-intervention and the intervention phase irrespective of the intervention on the four primary outcomes.

Outcome variables were log transformed because data were not normally distributed, except for observation days 0 and 1. Therefore, coefficients should be interpreted as a ratio, so that a coefficient of 1 means no difference. For all analyses, a random intercept on the “pneumonia episode level” was added to adjust for dependency of the repeated measures for each episode of pneumonia. To account for clustering of the pneumonia episodes within the participating homes, a random intercept was added at the nursing home level.

To assess possible differential intervention effects, we added interaction terms for group and intervention phase with (1) residents’ observed level of sleepiness and (2) death within 20 days or not. We did so because discomfort and symptoms may be different for residents observed awake or asleep and for residents who died within 20 days, because descriptive analyses of pre-intervention data showed an increase in discomfort in the six days preceding death [1] and because the EOLD-CAD has been developed for residents who are dying. When interactions were significant, we followed with subgroup analyses. We intended to perform subgroup analyses for residents treated with or without antibiotics, but these analyses were omitted as the proportion of residents who were not treated with antibiotics was too small [1]. With a sensitivity analysis, we examined effects when observations performed on the day of diagnosis were omitted, because the efficacy of some treatments, e.g., treatment with antibiotics, is expected only after the first day. Moreover, we examined whether results differed if only the first pneumonia episode per resident was included (exclusion of 24 non-first episodes). In exploratory analyses, we adjusted for observed sleepiness to examine if this altered the effect of the intervention, as decreasing consciousness could be a purpose of the intervention, e.g., with palliative sedation.

We adjusted all analyses for the a priori determined covariates sex, age, dementia severity (BANS-S score), type of dementia (Alzheimer’s or other), death within 20 days, and season (spring, summer, autumn, winter; because etiology and population may be different), and for baseline differences. The latter included resident characteristics and symptoms at baseline that differed within or between groups (type of dementia “mixed,” “other,” or “unknown,” full dependency in ADLs, dependency in dressing and eating, the number of comorbid diseases, hydration status, elevated body temperature, coughing, tachypnea, decreased alertness, and sputum production). We checked whether adjusting for a priori determined covariates alone altered results, but this was not the case. For covariates with missing data (a maximum of 7 % of the residents and 5 % of the observations), we applied multiple imputation with 20 imputations, after which data were pooled according to Rubin’s rules for analyses with data of all residents [24]. In analyses on subgroups of the data, adjusted analyses were performed on single imputed data for covariates (imputation of the variable mean for continuous variables, and of mode for dichotomous variables) rather than on multiple imputed data, after we had confirmed that differences in coefficients and confidence intervals between analyses performed on multiple or single imputation were only minor.

The statistical significance level was set to 0.05 for all analyses. Multilevel linear regressions and multiple imputations (Multivariate Imputation by Chained Equations (mice) package [25]) were performed using R software version 3.2.0 (2015) [26]. All other statistical analyses were performed using SPSS version 20.0 (IBM Corporation, New York, NY, 2013).

### Deviations from the initial protocol (research proposal)

Patients were included upon physician’s clinical diagnosis rather than upon evaluation of whether patients met McGeer’s surveillance criteria for pneumonia (requiring X-ray examination) or other lower respiratory infections [27], because we expected the additional check to discourage inclusion of patients (for example, if some symptoms were not assessed). However, 86 % of included patients met the criteria of McGeer for other lower respiratory infections. Further, because pre-intervention data showed no difference in the outcomes between patients treated with or without antibiotics, planned analyses of the effect of treating with antibiotics were not performed. The RDOS to assess respiratory distress was not part of the trial protocol but was found later and replaces the recording of a combination of items to address respiratory difficulty. The Reaction Level Scale was not used to assess coma, as it implies applying pain stimuli, which is not compatible with observations by the independent observers.

## Results

### Pneumonia episodes and observations

Between 1 January 2012 and 1 May 2015, the attending physicians included 464 episodes of pneumonia in the study and reported late 131 ”missed” episodes that met the inclusion criteria but were not reported in a timely manner. The incidence of pneumonia was 0.085 episode of pneumonia per resident per year. The nursing homes that participated in the study had an average of 100 beds (range 30–200) on one or more psychogeriatric wards.

**Fig. 2:**
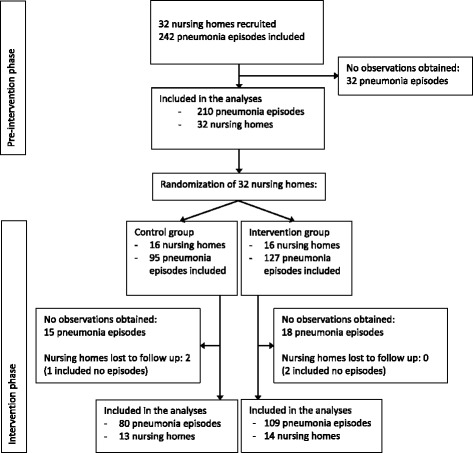
Trial profile

We obtained observational data for 399 episodes in 367 residents (18 residents participated twice, 4 participated three times, 2 participated four times). In the remaining 65 of the 464 episodes no observations took place, mostly due to organizational difficulties in scheduling of the observations, or because residents died soon after pneumonia diagnosis. The analyzed episodes included 210 (52.6 %) in the pre-intervention phase and 189 (47.4 %) in the intervention phase with 80 episodes in control homes and 109 episodes in intervention homes (Fig. 2).

### Characteristics of population and treatments

Overall, in 57.9 % of the episodes residents were female, and the mean age was 84.2 (SD 7.6) (Table 2). In most episodes (87.1 %), residents received antibiotic treatment. Physicians initiated treatment to relieve symptoms of pneumonia in 72.9 % of the episodes; antipyretics (46.7 %), oxygen (24.9 %), and morphine (16.0 %) were used most often. Despite cluster randomization of the homes, we found some differences between groups and phases (pre-intervention versus intervention) with regard to resident characteristics and symptoms of pneumonia (Table 2 and Additional file 1: Table S1), for which we adjusted in the analyses. Few differences were observed in symptom-relieving treatments between control and intervention homes and between the pre-intervention and intervention phase (Additional file 1: Table S2); in the pre-intervention phase, antipyretics were more common in intervention homes (55.0 % compared to 32.7 % in control homes), while in the intervention phase fewer residents received oxygen in intervention homes (20.6 %) compared to control homes (35.9 %).

**Table 2:** Resident characteristics, treatment, and mortality

		Pre-intervention phase		Intervention phase	
Characteristics	All (*n* = 399)	Control (*n* = 98)	Intervention (*n* = 112)	Control (*n* = 80)	Intervention (*n* = 109)
Demographics					
Female, % (*n*)	57.9 (231)	62.2 (61)	54.5 (61)	67.5 (54)^2^	50.5 (55)^2^
Age, mean (SD)	84.2 (7.6)	84.3 (7.3)	85.6 (6.3)^1^	83.7 (8.4)	83.3 (8.1)^1^
Length of stay, months, median (interquartile range)	18 (30)	17 (29)	23 (32) ^1^	22 (38) ^2^	12 (27) ^1, 2^
Dementia type, % (*n*)					
Alzheimer’s	37.8 (151)	34.7 (34)	36.6 (41)	41.2 (33)	39.4 (43)
Vascular	21.1 (84)	21.4 (21)	21.4 (24)	21.2 (17)	20.2 (22)
Mixed	15.3 (61)	12.2 (12)^2^	23.2 (26)^2, 1^	12.5 (10)	11.9 (13)^1^
Other	8.3 (33)	7.1 (7)	7.1 (8)	3.8 (3)^2^	13.8 (15)^2^
Unknown	17.6 (70)	24.5 (24)^2^	11.6 (13)^2^	21.2 (17)	14.7 (16)
Dementia severity, mean BANS-S score, mean (SD)	16.1 (4.6)	15.7 (4.7)	16.6 (4.8)	15.9 (4.6)	16.0 (4.5)
Full ADL dependency, % (*n*)	15.1 (55)	15.1 (13)	18.0 (18)^1^	20.5 (16)^2^	8.0 (8)^1, 2^
Dressing	44.5 (165)	45.3 (39)	52.5 (53)^1^	42.9 (33)	37.0 (37)^1^
Walking	35.8 (129)	38.1 (32)	39.8 (39)	37.2 (29)	29.0 (29)
Eating	22 (81)	21.6 (19)	28.4 (29)^1^	24.4 (19)	14.0 (14)^1^
Hydration status,* dehydrated, % (*n*)	31.2 (123)	39.8 (39)^1^	30.6 (34)	24.4 (19)^1^	29.0 (31)
Clinical judgment of illness severity (range 1–9), mean (SD)	5.3 (1.5)	5.3 (1.3)	5.6 (1.5)	5.2 (1.4)	5.3 (1.6)
Prognostic score (range 0–31), mean (SD)	14.2 (5.4)	14.7 (5.6)	14.0 (5.5)	14.2 (5.9)	13.9 (5.3)
Comfort goal, % (*n*)	62.2 (248)	62.2 (61)	63.4 (71)	67.5 (54)	56.9 (62)
Antibiotic treatment, % (*n*)	87.1 (344)	89.8 (88)	88.3 (89)	82.1 (64)	86.9 (93)
Observed non-pharmacological measures**	52.9 (211)	52.0 (51)	49.1 (55)	62.5 (50)	50.5 (55)
Death within 14 days	20.2 (80)	24.7 (24)	17.1 (19)	18.8 (15)	20.2 (22)
Death within 6 months	43.6 (154)	44.6 (41)	36.9 (41)	44.9 (31)	40.6 (41)

*Hydration status as judged by the attending physician; “mildly dehydrated,” “dehydrated,” and “severely dehydrated” were combined into “dehydrated”

**Observed non-pharmacological measures during at least one observation day between day 1 and day 5 from diagnosis

^1^Significant difference between pre-intervention and intervention phase in control homes and intervention homes (*p* < 0.05)

^2^Significant difference between control homes and intervention homes during pre-intervention and intervention phase (*p* < 0.05)

### The course of discomfort and symptoms

**Fig. 3:**
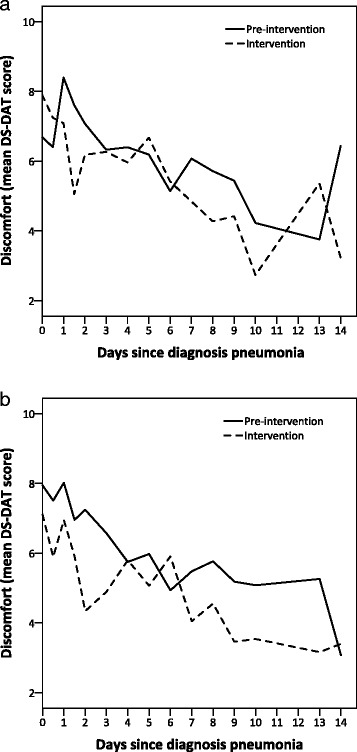
Mean discomfort (Discomfort Scale-Dementia of Alzheimer Type, *DS-DAT* range 0–27) per observation day in control homes (**a**) and intervention homes (**b**); pre-intervention phase compared to intervention phase. Range among groups (pre-intervention phase, intervention phase, intervention homes, control homes) in the number of observations per day from pneumonia diagnosis: Day 0 (first observation): 28–43; day 0 (second observation): 14–28; day 1 (first observation): 54–86; day 1 (second observation): 40–66; day 2: 55–94; day 3: 54–90; day 4: 52–79; day 5: 50–80; day 6: 50–84; day 7: 53–87; day 8: 49–79; day 9: 53–72; day 10: 12–24; day 13: 25–56; day 14: 12–21

The highest level of discomfort (DS-DAT) was measured on the day of diagnosis; from that point, levels declined to reach a stable level at around day 10 (Fig. 3). Comfort (EOLD-CAD), pain (PAINAD), and respiratory distress (RDOS) followed a similar course (Additional file 1: Figure S1).

### Effect of the intervention on discomfort and symptoms and secondary outcomes

Table 3 shows the effect of the intervention on the level of discomfort and symptoms from the diagnosis until 14 days later. The intervention was not effective in reducing the level of discomfort and symptoms in both unadjusted and adjusted models (adjusted, DS-DAT: 1.11 (95 % CI 0.93–1.31), reverse EOLD-CAD: 1.01 (95 % CI 0.98–1.05), PAINAD: 1.04 (95 % CI 0.93–1.15), RDOS: 1.11 (95 % CI 0.90–1.24)).

**Table 3:** Intervention effect on levels of discomfort, (lack of) comfort, pain, and respiratory distress

Level of suffering	Unadjusted analyses			Adjusted analyses^a^		
	Coefficient (95 % CI)		ICC	Coefficient (95 % CI)		ICC*
Discomfort (DS-DAT)	=	1.05 (0.88–1.25)	0.12	=	1.11 (0.93–1.31)	0.13
*Lack of* comfort (reverse EOLD-CAD)	=	1.01 (0.98–1.04)	0.10	=	1.01 (0.98–1.05)	0.14
Pain (PAINAD)	=	1.01 (0.90–1.12)	0.07	=	1.04 (0.93–1.15)	0.11
Respiratory distress (RDOS)	=	1.06 (0.94–1.19)	0.17	=	1.11 (0.99–1.24)	0.21

Discomfort (*DS-DAT* Discomfort Scale-Dementia of Alzheimer Type; range 0–27), (lack of) comfort (*EOLD-CAD*, End Of Life in Dementia-Comfort Assessment in Dying; range 14–42), pain (*PAINAD*, Pain Assessment in Advanced Dementia; range 0–10), respiratory distress (*RDOS,* Respiratory Distress Observation Scale; range 0–16)

Results from models without interaction terms of group and intervention phase with the level of observed sleepiness or death within 20 days

Analyses were performed on log-transformed data after which coefficients and confidence intervals were back-transformed. Therefore, coefficients should be interpreted as a ratio, so that a coefficient of 1 means no difference

*ICC*, intracluster correlation; *ICC for adjusted analyses determined with non-imputed data for covariates

*CI*, confidence interval

= No significance

^a^Adjusted for age, sex, season, severity of dementia (BANS-S score), type of dementia (Alzheimer’s or other), death within 20 days, and baseline differences (Table 2 resident characteristics, and Additional file 1 Table S1 symptoms fo pneumonia). Adjusted analyses are performed on multiply imputed data for covariates

Discomfort and symptoms were higher for residents who died within 20 days from the diagnosis of pneumonia and lower for residents who were observed asleep. A significant interaction between the intervention and death within 20 days for (lack of) comfort (EOLD-CAD) in both the unadjusted (*p* = 0.004) and adjusted models (*p* = 0.02) indicates that the intervention was the least effective for residents who died within 20 days from the diagnosis of pneumonia (Additional file 1: Table S3). In unadjusted analyses, the coefficient for residents who died within 20 days was 1.09 (>1 favors control group), and for those who did not die it was 0.98 (<1 favors intervention group).

The intervention effect was not different for residents who were observed awake or asleep; adding a correction for observed sleepiness to other adjustments did not notably affect the magnitude or the direction of the coefficients for the level of discomfort and symptoms (Additional file 1: Tables S3 and S4). Coefficients were similar with observations performed only after the first day and when limited to the first pneumonia episode per resident (Additional file 1: Table S5).

### Change of discomfort and symptoms over the study period

**Fig. 4:**
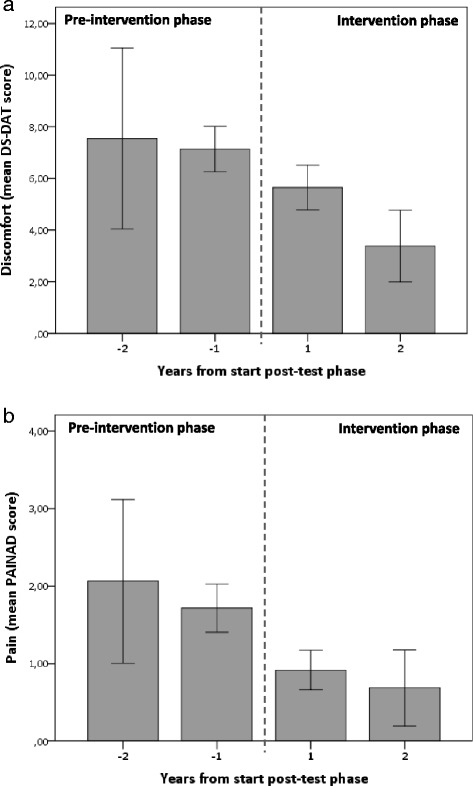
Mean DS-DAT score (**a**) and mean PAINAD score (**b**) (95 % confidence interval) on observation day 2 by year of data collection before and after the start of the intervention phase in both control and intervention homes. Range of the number of observations per period: −2 years: 14–16, –1 year: 136–140, 1 year: 105–109, 2 years: 35–36. *DS-DAT* Discomfort Scale-Dementia of Alzheimer Type; range 0–27, *PAINAD* Pain Assessment in Advanced Dementia; range 0–10

Discomfort and symptoms were significantly lower in the intervention phase compared to the pre-intervention phase, in both the control and the intervention homes: adjusted, DS-DAT: 0.93 (95 % CI 0.85–1.01), EOLD-CAD: 0.98 (95 % CI 0.97–1.00), PAINAD: 0.96 (95 % CI 0.91–1.01), RDOS: 0.92 (95 % CI 0.87–0.98) (Table 4, Additional file 1: Table S6). Although discomfort and symptoms decreased gradually over time, for lack of comfort, pain, and respiratory distress, the largest decrease was observed at the transition to the intervention phase (Fig. 4, Additional file 1: Figure S2).

**Table 4:** Level of discomfort, (lack of) comfort, pain, and respiratory distress in intervention phase versus pre-intervention phase for the control homes and the intervention homes together

Level of suffering	Unadjusted analyses			Adjusted analyses^a^		
	Coefficient (95 % CI)		ICC	Coefficient (95 % CI)		ICC*
Discomfort (DS-DAT)	+	0.90^*^ (0.82–0.98)	0.12	=	0.93 (0.85–1.01)	0.12
*Lack of* comfort (reverse EOLD-CAD)	+	0.98^*^ (0.96–0.99)	0.09	+	0.98^*^ (0.97–1.00)	0.13
Pain (PAINAD)	+	0.93^*^ (0.88–0.99)	0.07	=	0.96 (0.91–1.01)	0.10
Respiratory distress (RDOS)	+	0.91^*^ (0.86–0.97)	0.16	+	0.92^*^ (0.87–0.98)	0.18

Discomfort (*DS-DAT* Discomfort Scale-Dementia of Alzheimer Type; range 0–27), (lack of) comfort (*EOLD-CAD* End Of Life in Dementia-Comfort Assessment in Dying; range 14–42), pain (*PAINAD* Pain Assessment in Advanced Dementia; range 0–10), respiratory distress (*RDOS* Respiratory Distress Observation Scale; range 0–16)

Results from models without interaction terms of group and intervention phase with the level of observed sleepiness or death within 20 days

Analyses were performed on log-transformed data after which coefficients and confidence intervals were back-transformed. Therefore, coefficients should be interpreted as a ratio, so that a coefficient of 1 means no difference

*CI* confidence interval

*ICC* intracluster correlation; *ICC for adjusted analyses determined with non-imputed data for covariates

^*^ *p* < 0.05

+ Significant association that is interpreted in terms of a positive attribute (favors intervention phase: decrease in discomfort, lack of comfort, pain and respiratory distress)

= No significance

^a^Adjusted for age, sex, season, severity of dementia (BANS-S score), type of dementia (Alzheimer’s or other), death within 20 days and baseline differences (Table 2 resident characteristics, and Additional file 1 Table S1 symptoms fo pneumonia). Adjusted analyses are performed on multiply imputed data for covariates

## Discussion

We found that the physician practice guideline had no effect on discomfort and symptoms in a cluster randomized trial in 32 nursing homes. However, lack of comfort and respiratory distress gradually declined during the 3.5 years of observation in both the control and the intervention nursing homes.

### Discomfort and symptoms and effect of the intervention

Few studies have assessed discomfort in dementia residents using the DS-DAT [1, 3, 9, 28], but compared to these earlier studies of physician observations of residents with pneumonia [2] or in the last days of life [12], levels of discomfort in our study were particularly low. The rate of antibiotic administration was high compared to a previous Dutch observational study (van der Steen et al. [1]) in which 77 % of patients with dementia and pneumonia received antibiotic treatment compared to 87 % in our study; also, symptom-relieving treatments including opioids were provided more often. Nevertheless, the decrease of discomfort and symptoms during the study period in both control and intervention homes shows that there was room for improvement (mean DS-DAT score on day 3 in our 1996–1998 study: [2] 8.4; 2012–2015 pre-intervention phase: 6.5; intervention phase: 5.4).

Possible explanations for the lack of an overall intervention effect may relate to the characteristics of the practice guideline and how it was introduced to and applied by physicians. Although the practice guideline targeted the attending physicians, symptom-relieving treatments were not different in the intervention homes, which suggests that there was no change in physicians’ prescribing behavior. Data collected in the context of a process evaluation suggest that physicians who employed the practice guideline did not express a pressing need to use it because they felt that the recommendations matched their routines already [29]. Moreover, the observational instruments for pain and respiratory distress to monitor symptoms of pneumonia were not used regularly, which may indicate suboptimal adoption. Changing practice using interventions requires considerable effort, and guideline adherence may have been higher if more rigorous implementation strategies had been applied such as audit and feedback or multiple interactive meetings.

We found that the effects of the practice guideline were more favorable for residents who did not die within 20 days following pneumonia diagnosis. Perhaps there was more room for improvement for these residents, as physicians may have been less cognizant of comfort care’s importance for residents who are not expected to die from the pneumonia [28, 30, 31].

### Decrease of discomfort and symptoms over time

Discomfort and symptoms steadily decreased throughout 3.5 years of data collection. However, the largest change occurred with the transition to the intervention phase, which may be attributable to physician’s renewed attention to pneumonia symptoms such as pain and respiratory distress induced by both the practice guideline’s implementation in the intervention homes and the evaluation meeting in the control homes. Control homes may have attempted to do better out of a sense of competition. External factors, such as increased (media) attention for comfort and palliative care but also focus on this topic in education and further training, might account for the gradual decrease in discomfort and symptoms during this study. Moreover, the monitoring of discomfort and symptoms by external observers may have increased awareness of nursing staff members about the residents’ condition.

An effect of attention and increasing awareness is called a Hawthorne effect, and it concerns participants’ awareness of being studied that may impact their behavior. Studies addressing a Hawthorne effect are highly heterogeneous [32], but they share the characteristic that improvement of the outcome measure was due to attention or awareness and without an actual intervention, as is the case with, e.g., hand hygiene or appropriately prescribing antibiotics [33, 34]. Furthermore, participants are observed, or alternatively feel observed, such as with videotaping or participants being aware that their behavior is monitored. Social desirability considerations may (unconsciously or not) change behavior to be in line with the researchers’ expectations. In our study, the nursing staff’s feeling of being observed perhaps led to a greater focus on comfort or comfort measures which may have occurred gradually over time. In this way, collecting data may have been an effective intervention in itself, and this may hold as well for other populations.

### Strengths and limitations

This study is unique in the assessment of the outcomes on a regular (almost daily) basis by observers who were blinded to the intervention and to the residents’ treatments and condition. Furthermore, we used four different validated observational instruments to provide an overall picture of the effects of the practice guideline on discomfort and symptoms and its course over time. There are some limitations that should be acknowledged. First, 131 episodes fulfilled the inclusion criteria, but the attending physicians did not include them in a timely manner. With regard to residents’ sex, age, and antibiotic treatment, these episodes were not different from the episodes that were included. However, in the 65 of 464 cases that lacked observations, residents often died soon after diagnosis. These missing data were not at random, and the results are of course based on the residents who survived. Second, we chose outcome measures that capture pneumonia symptoms that are the most burdensome, but these do not necessarily cover all symptoms of pneumonia. The symptoms observed may have been caused by comorbid conditions or the terminal phase rather than by the pneumonia, but our aim was to assess the effect of a palliative intervention on symptoms after developing pneumonia regardless of causal mechanisms. Third, the proportion of residents not treated with antibiotics was too small (12.9 %) to perform subgroup analyses. Fourth, at times there was a substantial time period between observations of different patients by the same observer, and a number of different observers performed the observations. However, previous work showed that repeated observations with the DS-DAT are reliable when performed by the same observer after a number of months, and reliability is also acceptable among different observers [10]. Therefore, it is unlikely that the way discomfort was assessed changed substantially over time. Although it could take a while before they assessed the next patient, in view of previous findings, it is unlikely that the way discomfort was assessed changed over time. Lastly, we included 16 nursing homes per group, which was — according to the power analyses — adequate to detect relevant changes in the main outcome (DS-DAT) and the PAINAD, but low for the EOLD-CAD (20 per group needed). However, except for the first two observation days, the standard deviation in our data was lower than the standard deviation we used in the power calculation. This might be explained by higher levels and lower variability in comfort in residents who were not close to death, compared to retrospective assessment after death on which we based the power calculations (the instrument has been used prospectively successfully in residents expected to die, and we used it prospectively for all residents) [12].

## Conclusions

The developed practice guideline for optimal symptom relief had no effect on discomfort and symptoms in nursing home residents with dementia and pneumonia. Possible explanations for the lack of an intervention effect may be suboptimal implementation and also the fact that physicians often did not use the guideline because they felt that they already worked according to its recommendations. Moreover, an intervention can be more directive when there is a firmer scientific basis for symptom-relieving treatment. Discomfort was particularly low compared to previous research; moreover, it steadily decreased during the study period. Possible explanations for this favorable development are external factors such as increased attention in media and education or increased alertness due to the regular observer visits. Future studies may examine if an intervention directed at awareness of discomfort and regular observations by the nursing staff is more effective than a physician practice guideline.

## Abbreviations

DS-DAT, Discomfort Scale-Dementia of Alzheimer Type; EOLD-CAD, End Of Life in Dementia-Comfort Assessment in Dying; PAINAD, Pain Assessment in Advanced Dementia; RDOS, Respiratory Distress Observation Scale

## Additional file

Additional file 1: Table S1. Symptoms of pneumonia. **Table S2.** Treatments. **Figure S1**a–f. Mean comfort (*EOLD-CAD*: End Of Life in Dementia-Comfort Assessment in Dying; range 14–42) (a,b), pain (*PAINAD*: Pain Assessment in Advanced Dementia; range 0–10) (c,d), and shortness of breath (*RDOS*: Respiratory Distress Observation Scale; range 0–16) (e,f) per observation day in control homes (a,c,e) and intervention homes (b,d,f); pre-intervention phase compared to intervention phase. **Figure S2.** Mean EOLD-CAD score (95 % confidence interval) (a), mean RDOS score (95 % confidence interval) (b) on observation day 2 by year of data collection before and after the start of the intervention phase in both control and intervention homes. The range of the number of observations per period varied for the different outcomes: –2 years: 14–16, –1 year: 136–140, 1 year: 105–109, 2 years: 35–36. *EOLD-CAD*: End Of Life in Dementia – Comfort Assessment in Dying; (theoretical) range 14–42, *RDOS*: Respiratory Distress Observation Scale; range 0–16. **Table S3.** Secondary outcomes: testing of possible differential intervention effects by observed sleepiness and death within 20 days. **Table S4.** Exploratory analyses: intervention effect on levels of discomfort, (lack of) comfort, pain, and shortness of breath, and on change of the outcomes over time adjusted for the level of observed sleepiness. **Table S5.** Sensitivity analysis: intervention effect on levels of discomfort, (lack of) comfort, pain, and shortness of breath, and on change of the outcomes over time, without day 0. **Table S6.** Level of discomfort, (lack of) comfort, pain, and shortness of breath in intervention phase versus pre-intervention phase in control homes and intervention homes. (DOC 402 kb)
