# Supplementary material for: Improving comfort in people with dementia and pneumonia: a cluster randomized trial
Source: BMC Med. 2016 Aug 11;14:116. doi: 10.1186/s12916-016-0663-x (PMC4981997; doi:10.1186/s12916-016-0663-x)
Supplement: Additional file 1: Table S1. — Symptoms of pneumonia. Table S2. Treatments. Figure S1a–f. Mean comfort (EOLD-CAD: End Of Life in Dementia-Comfort Assessment in Dying; range 14–42) (a,b), pain (PAINAD: Pain Assessment in Advanced Dementia; range 0–10) (c,d), and shortness of breath (RDOS: Respiratory Distress Observation Scale; range 0–16) (e,f) per observation day in control homes (a,c,e) and intervention homes (b,d,f); pre-intervention phase compared to intervention phase. Figure S2. Mean EOLD-CAD score (95 % confidence interval) (a), mean RDOS score (95 % confidence interval) (b) on observation day 2 by year of data collection before and after the start of the intervention phase in both control and intervention homes. The range of the number of observations per period varied for the different outcomes: –2 years: 14–16, –1 year: 136–140, 1 year: 105–109, 2 years: 35–36. EOLD-CAD: End Of Life in Dementia – Comfort Assessment in Dying; (theoretical) range 14–42, RDOS: Respiratory Distress Observation Scale; range 0–16. Table S3. Secondary outcomes: testing of possible differential intervention effects by observed sleepiness and death within 20 days. Table S4. Exploratory analyses: intervention effect on levels of discomfort, (lack of) comfort, pain, and shortness of breath, and on change of the outcomes over time adjusted for the level of observed sleepiness. Table S5. Sensitivity analysis: intervention effect on levels of discomfort, (lack of) comfort, pain, and shortness of breath, and on change of the outcomes over time, without day 0. Table S6. Level of discomfort, (lack of) comfort, pain, and shortness of breath in intervention phase versus pre-intervention phase in control homes and intervention homes. (DOC 402 kb) [file 12916_2016_663_MOESM1_ESM.doc]

**Additional file**

**Additional tables and figures**

Table S1: Symptoms of pneumonia

|  | **Pre-intervention phase** | | **Intervention phase** | |
| --- | --- | --- | --- | --- |
| **Symptoms** | **Control**  **(n=98)** | **Intervention (n=112)** | **Control**  **(n=80)** | **Intervention (n=109)** |
| Number of symptoms (out of 13 listed below), mean (SD) | 5.4 (1.5) | 5.4 (2.0) | 5.2 (2.2) | 5.0 (2.1) |
| Symptoms pneumonia (new or acutely worse), % (n) |  |  |  |  |
| Auscultation abnormalities | 95.9 (93) | 92.7 (101) | 92.2 (71) | 93.3 (98) |
| General malaise | 84.5 (82) | 78.9 (86) | 79.5 (62) | 86.4 (89) |
| Elevated body temperature | 76.3 (74) | 74.3 (81)^a^ | 76.9 (60)^b^ | 59.0 (62)^ab^ |
| Coughing | 71.4 (70) | 64.9 (72) | 59.0 (46)^b^ | 75.5^b^ |
| Dyspnea | 58.8 (57) | 56.9 (62) | 50.6 (39) | 50.0 (53) |
| Tachypnea | 53.6 (52)^a^ | 57.4 (62) | 72.0 (54)^ab^ | 48.5 (49)^b^ |
| Decreased alertness | 36.7 (36)^a^ | 40.5 (45)^a^ | 19.5 (15)^a^ | 26.7 (28)^a^ |
| Tachycardia | 36.0 (32) | 34.9 (38) | 48.0 (36) | 37.4 (37) |
| Sputum production | 21.3 (19)^b^ | 35.2 (37)^b^ | 24.0 (18) | 26.9 (28) |
| Orthopnea | 7.4 (7) | 13.2 (14) | 11.8 (9) | 8.8 (9) |
| Lowered body temperature | 4.3 (4) | 2.8 (3) | 1.3 (1) | 5.1 (5) |
| Pleuritic chest pain | 2.2 (2) | 4.2 (4) | 1.4 (1) | 0.0 (0) |
| Cheyne-Stokes respiration | 3.1 (3) | 1.8 (2) | 1.3 (1) | 2.0 (2) |

a: Significant difference between pre-intervention and intervention phase (p<0.05)

b: Significant difference during pre-intervention or intervention phase (p<0.05)

Table S2: Treatments

|  | **Pre-intervention phase** | | **Intervention phase** | |
| --- | --- | --- | --- | --- |
| **Treatments** | **Control**  **(n=98)** | **Intervention (n=112)** | **Control**  **(n=80)** | **Intervention (n=109)** |
| Antibiotics | 89.8 (88) | 88.3 (89) | 82.1 (64) | 86.9 (93) |
| Intravenous rehydration | 1.0 (1) | 0.0 (0) | 0.0 (0) | 0.0 (0) |
| Rehydration by hypodermoclysis | 6.1 (6) | 2.7 (3) | 3.8 (3) | 2.8 (3) |
| Rehydration by encouraging fluid intake | 36.7 (36) | 45.9 (51)^a^ | 33.3 (26) | 32.7 (35)^a^ |
| Observed non-pharmacological measures^‡^ | 52.0 (51) | 49.1 (55) | 62.5 (50) | 55.5 (50) |
|  |  |  |  |  |
| Treatments to relieve symptoms of pneumonia |  |  |  |  |
| Antipyretics | 32.7 (32)^b^ | 55.0 (61)^b^ | 41.0 (32) | 55.1 (59) |
| Oxygen | 24.5 (24) | 21.6 (24) | 35.9 (28)^b^ | 20.6 (22)^b^ |
| Opioids | 14.3 (14) | 16.2 (18) | 14.1 (11) | 18.7 (20) |
| Hypnotics | 4.1 (4) | 4.5 (5) | 3.8 (3) | 3.7 (4) |
| Bronchodilators | 17.3 (17) | 10.8 (12) | 10.3 (8) | 13.1 (4) |
| Corticosteroids | 3.1 (3) | 3.6 (4) | 6.4 (5) | 4.7 (5) |
| Anticholinergics | 3.1 (3) | 0.9 (1) | 1.3 (1) | 0.0 (0) |
| Antipsychotics | 1.0 (1) | 2.7 (3) | 2.6 (2) | 0.9 (1) |
| Other | 4.1 (4) | 5.4 (6) | 2.6 (2) | 1.9 (2) |
| Any pharmacological treatment to relieve symptoms | 66.3 (65) | 75.9 (85) | 71.2 (57) | 77.1 (84) |
|  |  |  |  |  |
| Number treatments to relieve symptoms, mean (SD) | 1.0 (0.98) | 1.2 (1.1) | 1.2 (0.92) | 1.2 (1.0) |

^‡^observed non-pharmacological measures during at least one observation day between day 1 and day 5 from diagnosis

a: Significant difference between pre-intervention and intervention phase (p<0.05)

b: Significant difference during pre-intervention or intervention phase (p<0.05)

*
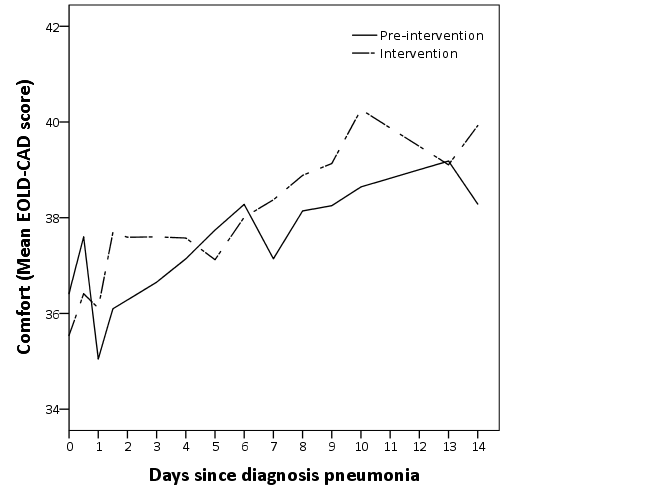

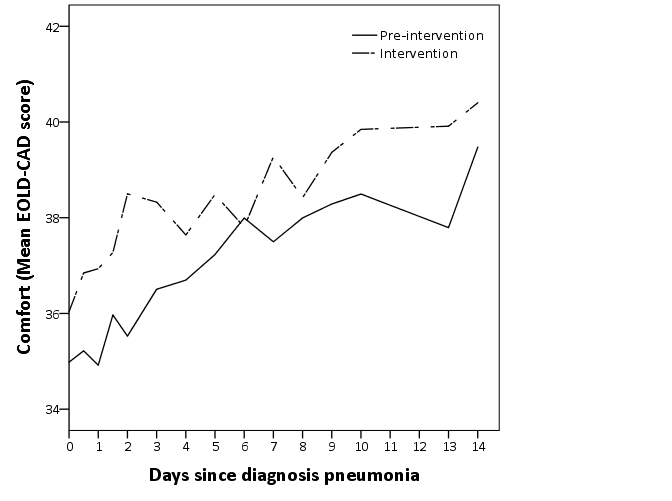
*

a Comfort in control homes b Comfort in intervention homes


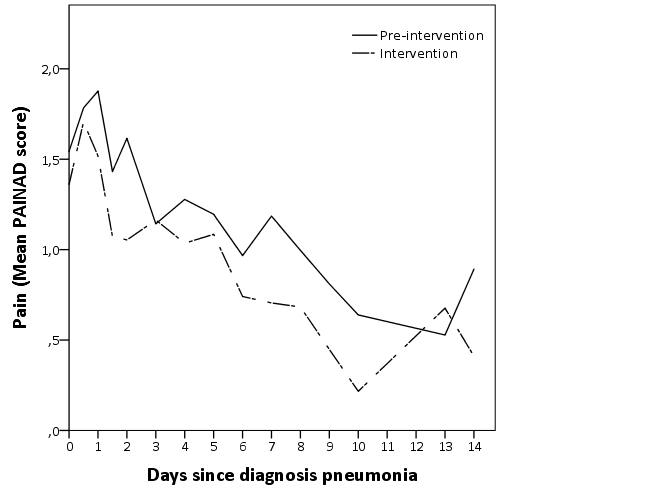

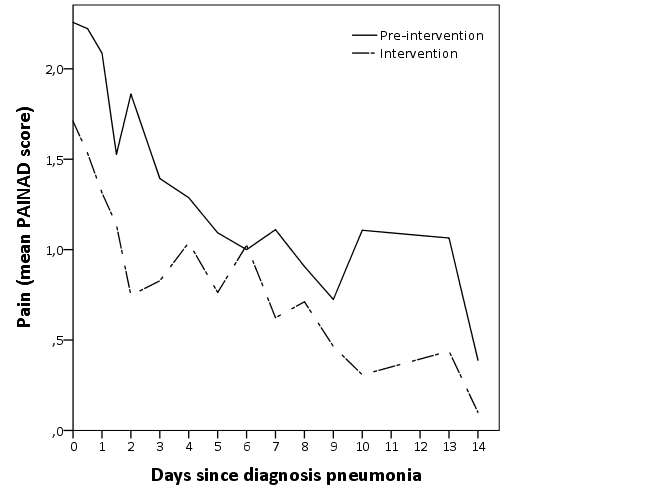


c Pain in control homes d Pain in intervention homes


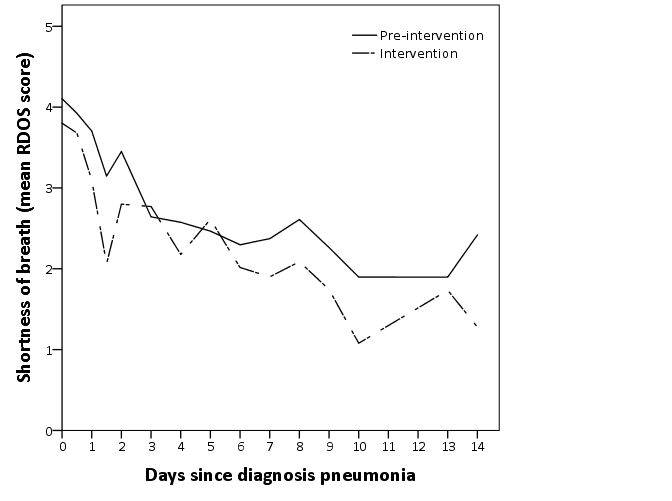

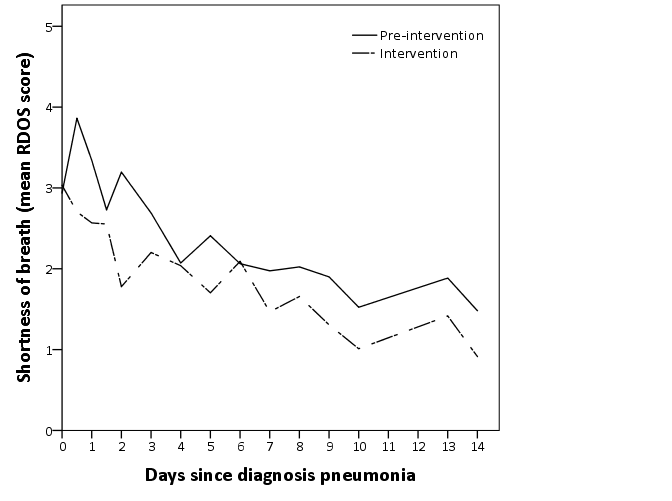


e Shortness of breath in control homes f Shortness of breath in intervention homes

Figure S1a-f. Mean comfort (EOLD-CAD: End Of Life in Dementia – Comfort Assessment in Dying; range 14-42) (a,b), pain (PAINAD: Pain Assessment In Advanced Dementia; range 0-10) (c,d), and shortness of breath (RDOS: Respiratory Distress Observation Scale; range 0-16) (e,f) per observation day in control homes (a,c,e) and intervention homes (b,d,f); pre-intervention phase compared to intervention phase*.*

*
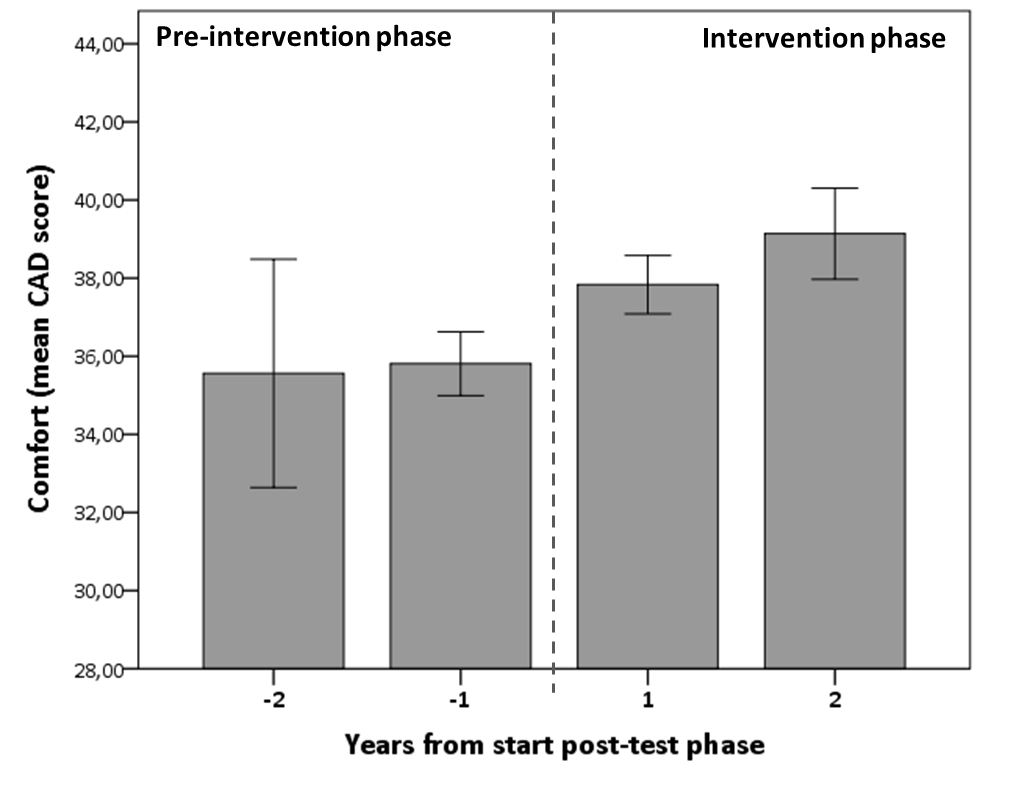
*

a

*
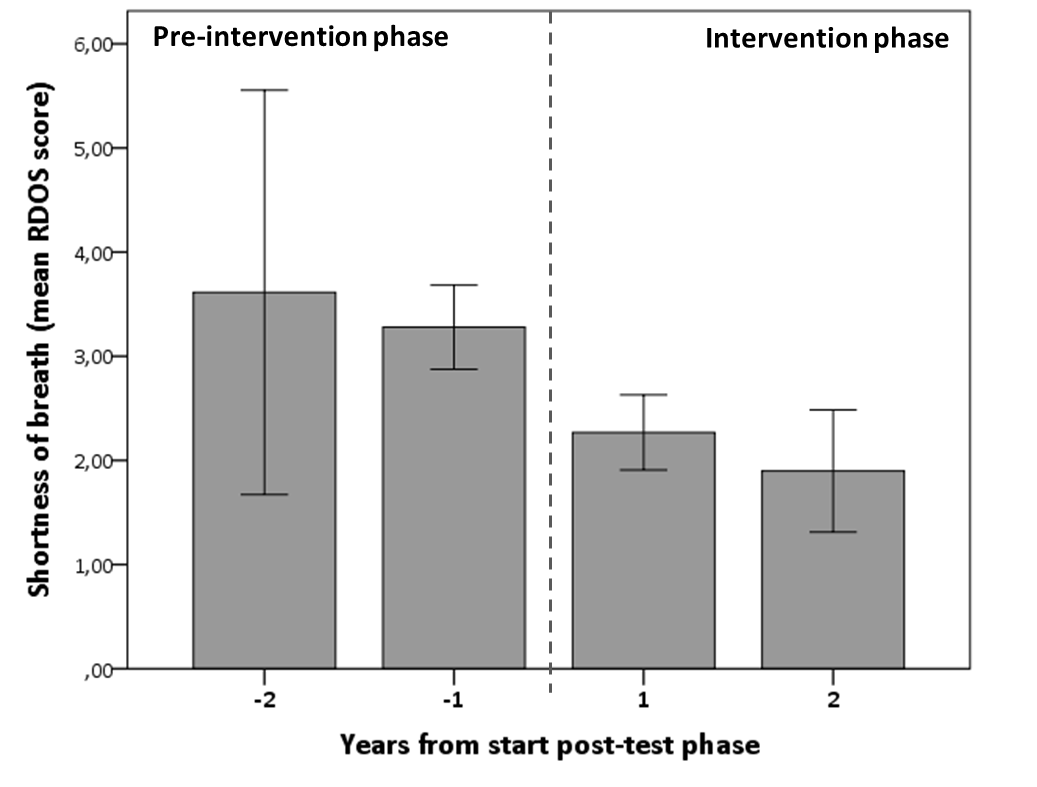
*

b

Figure S2: Mean EOLD-CAD score (95% confidence interval) (a), mean PAINAD score (95% confidence interval), (b) and mean RDOS score (95% confidence interval), (b) on observation day 2 by year of data collection before and after the start of the intervention phase in both control and intervention homes. The range of the number of observations per period varied for the different outcomes: -2 years: 14-16, -1 year: 136-140, 1 year: 105-109, 2 years: 35-36. EOLD-CAD: End Of Life in Dementia – Comfort Assessment in Dying; (theoretical) range 14-42, Respiratory Distress Observation Scale; range 0-16*.*

Table S3: secondary outcomes - testing of possible differential intervention effects by observed sleepiness and dead within 20 days*.*

| Interaction with observed sleepiness | Unadjusted analyses | | Adjusted analyses^1^ | |
| --- | --- | --- | --- | --- |
|  | Coefficient (95% CI) | | Coefficient (95% CI) | |
| Discomfort (DS-DAT) | = | 1.01 (0.96 - 1.06) | = | 1.01 (0.96 - 1.06) |
| *Lack of* comfort (EOLD-CAD) | = | 1.00 (0.99 - 1.01) | = | 1.00 (0.99 - 1.00) |
| Pain (PAINAD) | = | 0.99 (0.96 - 1.02) | = | 0.99 (0.96 - 1.02) |
| Shortness of breath (RDOS) | = | 0.98 (0.94 - 1.01) | = | 0.98 (0.94 - 1.03) |
| Interaction with death within 20 days | Unadjusted analyses | | Adjusted analyses^1^ | |
|  | Coefficient (95% CI) | | Coefficient (95% CI) | |
| Discomfort (DS-DAT) | – | 1.58^*^ (1.05 - 2.38) | = | 1.41 (0.94 - 2.12) |
| *Lack of* comfort (EOLD-CAD) | – | 1.12^*^ (1.04 - 1.21) | – | 1.10^*^ (1.02 - 1.18) |
| Pain (PAINAD) | – | 1.38^*^ (1.06 - 1.79) | = | 1.26 (0.97 - 1.63) |
| Shortness of breath (RDOS) | = | 1.02 (0.78 - 1.34) | = | 0.98 (0.75 - 1.28) |

Discomfort (DS-DAT: Discomfort Scale – Dementia of Alzheimer Type; range 0-27), (lack of) comfort (EOLD-CAD: End Of Life in Dementia – Comfort Assessment in Dying; range 14-42), pain (PAINAD: Pain Assessment In Advanced Dementia; range 0-10), shortness of breath (RDOS: Respiratory Distress Observation Scale; range 0-16).

Analyses were performed on log-transformed data after which coefficients and confidence intervals were back-transformed. Therefore, coefficients should be interpreted as a ratio, so that a coefficient of 1 means no difference.

CI: confidence interval

* p < 0.05

- significant interaction with the intervention that is interpreted in terms of a negative attribute (the intervention was less effective for residents who died within 20 days than for those who did not die within 20 days).

= no significant interaction

^1^ adjusted for age, sex, season, severity of dementia (BANS-S score), type of dementia (Alzheimer’s or other), death within 20 days and baseline differences (table 1 characteristics and table 2 symptoms of pneumonia)). Adjusted analyses are performed on multiply imputed data for covariates.

Table S4: exploratory analyses: intervention effect on levels of discomfort, (lack of) comfort, pain and shortness of breath, and on change of the outcomes over time adjusted for the level of observed sleepiness

| Level of suffering | Unadjusted analyses | | Adjusted analyses^1^ | |
| --- | --- | --- | --- | --- |
|  | Coefficient (95% CI) | | Coefficient (95% CI) | |
| Discomfort (DS-DAT) | = | 1.04 (0.87 - 1.25) | = | 1.10 (0.93 - 1.31) |
| *Lack of* comfort (reverse EOLD-CAD) | = | 1.01 (0.97 - 1.04) | = | 1.01 (0.98 - 1.05) |
| Pain (PAINAD) | = | 1.00 (0.90 - 1.12) | = | 1.04 (0.93 - 1.15) |
| Shortness of breath (RDOS) | = | 1.06 (0.94 - 1.19) | = | 1.11 (0.99 - 1.24) |

Discomfort (DS-DAT: Discomfort Scale – Dementia of Alzheimer Type; range 0-27), (lack of) comfort (EOLD-CAD: End Of Life in Dementia – Comfort Assessment in Dying; range 14-42), pain (PAINAD: Pain Assessment In Advanced Dementia; range 0-10), shortness of breath (RDOS: Respiratory Distress Observation Scale; range 0-16)

Models without interaction terms of group and invention phase with the level of observed sleepiness or death within 20 days

Analyses were performed on log-transformed data after which coefficients and confidence intervals were back-transformed. Therefore. coefficients should be interpreted as a ratio.

CI: confidence interval

= no significance

^1^ adjusted for age. sex. season. severity of dementia (BANS-S score). type of dementia (Alzheimer’s or other). death within 20 days and baseline differences (table 1 characteristics and table 2 symptoms of pneumonia)). Adjusted analyses are performed on multiply imputed data for BANS-S score and type of dementia.

Table S5: sensitivity analysis: intervention effect on levels of discomfort, (lack of) comfort, pain and shortness of breath, and on change of the outcomes over time, without day 0

| Level of suffering | Unadjusted analyses | | Adjusted analyses^1^ | |
| --- | --- | --- | --- | --- |
|  | Coefficient (95% CI) | | Coefficient (95% CI) | |
| Discomfort (DS-DAT) | = | 1.07 (0.89 - 1.28) | = | 1.16 (0.97 - 1.38) |
| *Lack of* comfort (reverse EOLD-CAD) | = | 1.01 (0.97 - 1.04) | = | 0.99 (0.98 - 1.05) |
| Pain (PAINAD) | = | 1.00 (0.89 - 1.12) | = | 1.04 (0.93 - 1.17) |
| Shortness of breath (RDOS) | = | 1.05 (0.93 - 1.19) | = | 1.09 (0.97 - 1.23) |

Discomfort (DS-DAT: Discomfort Scale – Dementia of Alzheimer Type; range 0-27), (lack of) comfort (EOLD-CAD: End Of Life in Dementia – Comfort Assessment in Dying; range 14-42), pain (PAINAD: Pain Assessment In Advanced Dementia; range 0-10), shortness of breath (RDOS: Respiratory Distress Observation Scale; range 0-16).

Results from models without interaction terms of group and invention phase with the level of observed sleepiness or death within 20 days

Analyses were performed on log-transformed data after which coefficients and confidence intervals were back-transformed. Therefore, coefficients should be interpreted as a ratio, so that a coefficient of 1 means no difference.

CI: confidence interval

= no significance

^1^ adjusted for age, sex, season, severity of dementia (BANS-S score), type of dementia (Alzheimer’s or other), death within 20 days and baseline differences (table 1 characteristics and table 2 symptoms of pneumonia)). Adjusted analyses are performed on single imputed data (imputation of mean for continuous variables; and of mode for dichotomous variables).

Table S6: the level of discomfort, (lack of) comfort, pain and shortness of breath in intervention phase vs pre-intervention phase in control homes and intervention homes

| **Control homes** | Unadjusted analyses | | Adjusted analyses^1^ | |
| --- | --- | --- | --- | --- |
|  | Coefficient (95% CI) | | Coefficient (95% CI) | |
| Discomfort (DS-DAT) | + | 0.87^*^ (0.76 - 0.99) | = | 0.84 (0.73 - 1.07) |
| *Lack of* comfort (reverse EOLD-CAD) | + | 0.97^*^ (0.95 - 1.00) | = | 0.98 (0.95 - 1.00) |
| Pain (PAINAD) | = | 0.93 (0.86 - 1.01) | = | 0.93 (0.85 - 1.02) |
| Shortness of breath (RDOS) | + | 0.88^*^ (0.80 - 0.96) | + | 0.87^*^ (0.79 - 0.95) |
| **Intervention homes** | Unadjusted analyses | | Adjusted analyses^1^ | |
|  | Coefficient (95% CI) | | Coefficient (95% CI) | |
| Discomfort (DS-DAT) | = | 0.91 (0.81 - 1.02) | = | 0.95 (0.84 - 1.07) |
| *Lack of* comfort (reverse EOLD-CAD) | = | 0.98 (0.96 - 1.00) | = | 0.99 (0.97 - 1.02) |
| Pain (PAINAD) | = | 0.93 (0.87 - 1.01) | = | 0.99 (0.92 - 1.08) |
| Shortness of breath (RDOS) | = | 0.93 (0.86 - 1.01) | = | 0.97 (0.90 - 1.05) |

Discomfort (DS-DAT: Discomfort Scale – Dementia of Alzheimer Type; range 0-27), (lack of) comfort (EOLD-CAD: End Of Life in Dementia – Comfort Assessment in Dying; range 14-42), pain (PAINAD: Pain Assessment In Advanced Dementia; range 0-10), shortness of breath (RDOS: Respiratory Distress Observation Scale; range 0-16).

Results from models without interaction terms of group and invention phase with the level of observed sleepiness or death within 20 days

Analyses were performed on log-transformed data after which coefficients and confidence intervals were back-transformed. Therefore, coefficients should be interpreted as a ratio, so that a coefficient of 1 means no difference.

CI: confidence interval

* p < 0.05

+ significance association that is interpreted in terms of a positive attribute (favours intervention phase: decrease in discomfort, lack of comfort, pain and shortness of breath)

= no significance

^1^ adjusted for age, sex, season, severity of dementia (BANS-S score), type of dementia (Alzheimer’s or other), death within 20 days and baseline differences (table 1 characteristics and table 2 symptoms of pneumonia)). Adjusted analyses are performed single imputed data (imputation of mean for continuous variables; and of mode for dichotomous variables).
